# Diagnostic Performance of Mini Parasep^®^ for the Detection of Intestinal Parasitic Infections: A Systematic Review and Meta-Analysis

**DOI:** 10.3390/medicina62081464

**Published:** 2026-07-28

**Authors:** Jurairat Jongthawin, Aongart Mahittikorn, Kinley Wangdi, Frederick Ramirez Masangkay, Manas Kotepui

**Affiliations:** 1 Faculty of Medicine, Mahasarakham University, Maha Sarakham 44000, Thailand; jurairat.j@msu.ac.th; 2 Department of Protozoology, Faculty of Tropical Medicine, Mahidol University, Bangkok 10400, Thailand; 3 HEAL Global Research Center, Health Research Institute, Faculty of Health, University of Canberra, Bruce, ACT 2617, Australia; kinley.wangdi@canberra.edu.au; 4 National Centre for Epidemiology and Population Health, College of Law, Governance and Policy, Australian National University, Acton, ACT 2601, Australia; 5 Department of Medical Technology, Faculty of Pharmacy, University of Santo Tomas, Manila 1008, Philippines; frmasangkay@ust.edu.ph; 6 Research Center for the Natural and Applied Sciences, University of Santo Tomas, Manila 1008, Philippines; 7 Medical Technology Program, Faculty of Science, Nakhon Phanom University, Nakhon Phanom 48000, Thailand

**Keywords:** Mini Parasep^®^, fecal parasite concentrator, intestinal parasitic infections, diagnostic accuracy, sensitivity, systematic review, meta-analysis

## Abstract

*Background and Objectives:* Accurate diagnosis of intestinal parasitic infections remains essential for patient management, surveillance, and control programs. The Mini Parasep^®^ and Mini Parasep^®^ Solvent-Free (SF) fecal parasite concentrators were developed to improve laboratory safety and simplify stool processing; however, their diagnostic performance has not been comprehensively synthesized. This systematic review and meta-analysis evaluated the diagnostic sensitivity of Mini Parasep^®^ for detecting intestinal parasites in human stool specimens. *Materials and Methods:* A systematic literature search was conducted in Ovid, Embase, Scopus, PubMed, Nursing & Allied Health Premium, and Google Scholar from database inception to the final search date. Studies evaluating Mini Parasep^®^ or Mini Parasep^®^ SF in human stool samples and reporting sufficient data to calculate diagnostic sensitivity were eligible. A composite reference standard, defined as positivity by any diagnostic method used within each study, was accepted as the reference standard. Pooled sensitivity estimates and 95% confidence intervals (CIs) were calculated using random-effects models. Subgroup analyses were performed according to parasite species, study design, geographic region, country, and participant characteristics. *Results:* Eleven studies met the inclusion criteria. Overall, Mini Parasep^®^ demonstrated good sensitivity for detecting any intestinal parasitic infection, with a pooled sensitivity of 82.4% (95% CI: 61.0–93.4%, *I*^2^ = 95.3%, 6 studies). Among pathogenic protozoa, pooled sensitivities were 78.9% (95% CI: 39.3–95.6%, *I*^2^ = 43.7%, 4 studies) for *Entamoeba histolytica*, 76.3% (95% CI: 34.8–95.1%, *I*^2^ = 71.1%, 4 studies) for *Giardia duodenalis*, and 74.4% (95% CI: 60.4–84.7%, *I*^2^ = 0%, 2 studies) for *Blastocystis* spp. Among soil-transmitted helminths, sensitivity was highest for *Ascaris lumbricoides* (92.8%; 95% CI: 88.4–95.7%, *I*^2^ = 0%, 2 studies), followed by *Strongyloides stercoralis* (73.7%; 95% CI: 50.2–88.6%, *I*^2^ = 0%; 3 studies), hookworm (53.6%; 95% CI: 33.3–72.8%, *I*^2^ = 23.1%, 5 studies), and *Trichuris trichiura* (39.4%; 95% CI: 8.6–81.8%, *I*^2^ = 74.8%; 3 studies). High sensitivities were observed for *Taenia* spp. (89.0%; 95% CI: 74.0–95.8%, *I*^2^ = 0%; 3 studies) and *Schistosoma mansoni* (92.8%; 95% CI: 86.9–96.1%, *I*^2^ = 0%, 2 studies), whereas sensitivity for *Opisthorchis viverrini* was moderate (56.8%; 95% CI: 35.1–76.1%, *I*^2^ = 88.8%, 7 studies). High heterogeneity was observed for several parasite-specific analyses. *Conclusions:* Mini Parasep^®^ demonstrates good overall diagnostic sensitivity and performs particularly well for *A. lumbricoides*, *S. mansoni*, and *Taenia* spp. However, diagnostic performance varies substantially across parasite species, with lower sensitivity observed for hookworm, *T. trichiura*, and *O. viverrini*. Mini Parasep^®^ represents a practical alternative to conventional concentration techniques for routine stool examination, although complementary diagnostic approaches may be required when detecting low-intensity infections or parasites with lower recovery rates.

## 1. Introduction

Intestinal parasitic infections (IPIs) affect an estimated 1.5 billion people globally, representing a critical public health challenge, particularly across tropical and subtropical regions where socioeconomic constraints and inadequate sanitation facilitate transmission [1,2]. These infections comprise a diverse spectrum of pathogens that cause substantial morbidity, ranging from chronic diarrhea and malnutrition to growth stunting in children and cognitive impairment [3]. The major pathogens include pathogenic protozoa (*Entamoeba histolytica*, *Giardia duodenalis*, and potentially pathogenic include *Blastocystis* spp.), soil-transmitted helminths (STHs) (*Ascaris lumbricoides*, hookworms, *Trichuris trichiura*, and *Strongyloides stercoralis*), tapeworms (*Taenia* spp.), and flukes (*Opisthorchis viverrini* and *Schistosoma* spp.) [4,5]. Despite significant progress in preventive chemotherapy and integrated control programs, intestinal parasitic infections remain highly endemic in many regions and continue to pose major challenges for disease surveillance, patient management, and monitoring of intervention effectiveness [6].

In routine laboratory settings, manual copromicroscopy remains the primary diagnostic approach; however, conventional techniques possess well-documented analytical and practical shortcomings. Direct wet smears are rapid but suffer from low analytical sensitivity, often missing light infections. The Kato-Katz thick smear, while widely used for mapping STHs, fails to preserve protozoan parasites and shows a sharp drop in sensitivity at low worm burdens [7]. Alternatively, the traditional formalin-ethyl acetate concentration technique (FECT) achieves high organism recovery by clearing excessive debris, but its open, multi-step execution is time-consuming and regularly exposes laboratory personnel to hazardous chemical solvents [8,9].

To mitigate these operational biosafety risks and streamline diagnostic workflows, commercial enclosed concentration systems such as the Mini Parasep^®^ and Mini Parasep^®^ Solvent-Free (SF) fecal parasite concentrators were introduced. These single-use devices process stool specimens through an integrated filtration matrix, clearing obscuring debris without open chemical exposure. Over the past decade, Mini Parasep^®^ has been evaluated in various epidemiological settings for the detection of intestinal protozoa and helminths. Studies conducted in many countries have reported promising results, with high diagnostic sensitivity observed for certain parasites, including *A. lumbricoides*, *S. mansoni*, and *Taenia* spp. [8,10,11].

However, considerable variability has been reported across studies and parasite species. For example, while sensitivities exceeding 90% have been reported for *A. lumbricoides* and *S. mansoni*, lower or highly heterogeneous sensitivities have been observed for hookworm, *T. trichiura*, and *O. viverrini* [10,11,12]. Comparative evaluations show that the Kato-Katz method can outperform Mini Parasep^®^ SF in detecting *T. trichiura*, while the McMaster technique has demonstrated greater sensitivity in detecting hookworm burdens [10]. Furthermore, in low-intensity transmission settings, the diagnostic sensitivity of the Mini Parasep^®^ SF system has been shown to fall to 35.1% for *O. viverrini*, trailing dramatically behind formalin-ethyl acetate concentration methods [12]. Differences in parasite biology, infection intensity, study design, participant characteristics, specimen preservation, and comparator methods may contribute to the heterogeneity of these findings. Consequently, the overall diagnostic utility of Mini Parasep^®^ remains incompletely understood. Despite the growing body of literature evaluating Mini Parasep^®^, the available evidence has not been comprehensively synthesized. To our knowledge, no systematic review and meta-analysis has quantitatively assessed the diagnostic sensitivity of Mini Parasep^®^ and Mini Parasep^®^ Solvent-Free fecal parasite concentrators across multiple intestinal parasite species using a composite reference standard. This evidence gap hinders the development of evidence-based guidelines for laboratory diagnosis and limits the ability of endemic countries to make informed decisions about adopting this technology in routine practice.

Therefore, this systematic review and meta-analysis aimed to evaluate the diagnostic sensitivity of Mini Parasep^®^ and Mini Parasep^®^ Solvent-Free fecal parasite concentrators for detecting intestinal parasites in human stool specimens. In addition, we assessed diagnostic performance according to parasite species, study design, geographic region, country, and participant characteristics to provide a comprehensive evaluation of the role of Mini Parasep^®^ in contemporary parasitological diagnosis.

## 2. Methods

### 2.1. Protocol and Registration

**Figure 1:**
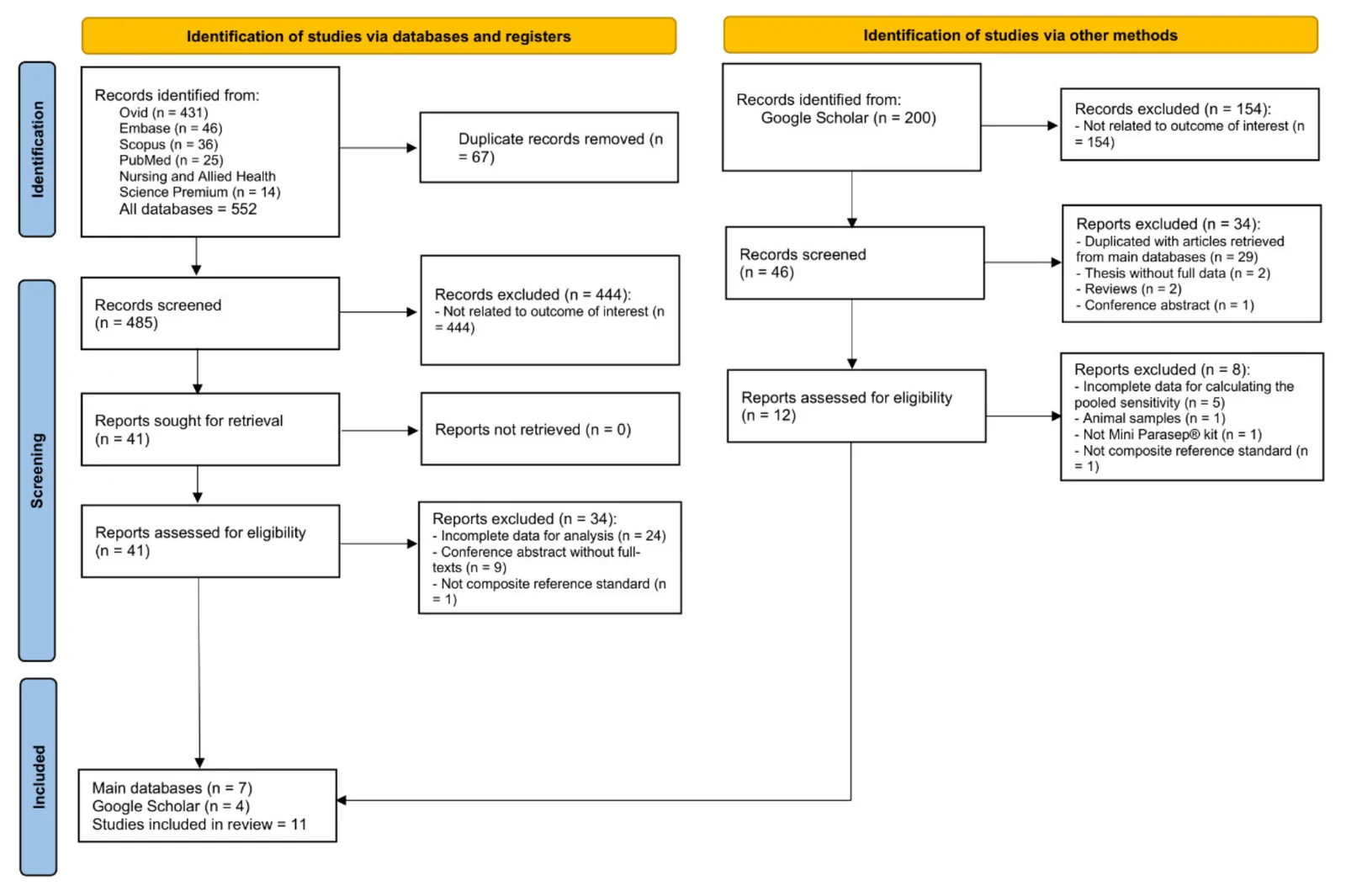
The PRISMA 2020 flow diagram illustrates the study selection process.

This systematic review and meta-analysis was conducted to evaluate the diagnostic sensitivity of the Mini Parasep^®^ fecal parasite concentrator for the detection of intestinal parasites in human stool specimens. The review followed the Preferred Reporting Items for Systematic Reviews and Meta-Analyses (PRISMA 2020) guidelines. The PRISMA Flow Diagram was shown in Figure 1. The protocol was registered at PROSPERO (CRD420261376930).

### 2.2. Outcomes

The primary outcome of this systematic review and meta-analysis was the pooled diagnostic sensitivity of Mini Parasep^®^ for detecting intestinal parasites. Analyses were conducted for any intestinal parasite infection, protozoa including *E. histolytica*, *G. duodenalis*, and *Blastocystis* spp.; soil-transmitted helminths (STHs) including *A. lumbricoides*, hookworms, *T. trichiura*, and *S. stercoralis*; tapeworms including *Taenia* spp.; and flukes including *O. viverrini* and *S. mansoni*.

### 2.3. Eligibility Criteria

Studies were eligible for inclusion if they evaluated the diagnostic sensitivity of Mini Parasep^®^ or Mini Parasep^®^ SF fecal parasite concentrator in human stool samples and reported sufficient data to calculate diagnostic sensitivity. Studies were required to use a composite reference standard, whereby a participant was considered positive if at least one diagnostic method detected the parasite of interest. Original research articles published in peer-reviewed journals were included regardless of geographic location or study population. Studies were excluded if they were review articles, conference abstracts without full-text availability, editorials, letters, case reports, or studies lacking sufficient data for diagnostic accuracy analysis. Studies that did not employ a composite reference standard or were conducted using non-human samples were also excluded.

### 2.4. Search Strategy

A comprehensive literature search was conducted in Ovid, Embase, Scopus, PubMed, and Nursing & Allied Health Premium from database inception to 19 May 2026. Additional records were identified through Google Scholar searches. The search strategy incorporated controlled vocabulary terms and free-text keywords related to the Mini Parasep^®^ fecal parasite concentrator, including “Mini Parasep^®^”, “Mini Parasep^®^ Solvent Free”, “Mini Parasep”, “Mini Parasep SF”, “Mini Parasep^®^ SF Faecal Parasite Concentrator”, and “Mini Parasep^®^ SF Stool Concentrator Kit”. Search strategies were tailored to the indexing terms and search interfaces of individual databases. Full search strategies for all databases are provided in Table S1.

### 2.5. Study Selection

All retrieved records were imported into a reference management system (EndNote version 21.0, Philadelphia, PA, USA), and duplicate records were removed prior to screening. Titles and abstracts were independently screened by two authors (M.K., A.M.) to identify potentially relevant studies. Full-text articles were subsequently assessed for eligibility according to the predefined inclusion and exclusion criteria. Any disagreements regarding study inclusion were resolved through discussion and consensus.

### 2.6. Data Extraction

Data were independently extracted by two authors (M.K., J.J.) using a standardized data extraction form, with discrepancies resolved by the third author (A.M.). Information collected from each study included the first author, year of publication, country, study design, study population, sample size, type of intestinal parasite investigated, index test characteristics, reference standard, and diagnostic accuracy outcomes. For each parasite species, the number of positive cases detected by Mini Parasep^®^ and the total number of positive cases identified by the composite reference standard were extracted. Where studies reported results for multiple parasite species, data were extracted separately for each species to permit parasite-specific meta-analyses.

### 2.7. Reference Standard

Because no universally accepted gold standard exists for diagnosing most intestinal parasitic infections, the composite reference standard used in the original studies was adopted for this review. In all included studies, a participant was classified as positive if at least one diagnostic method used in the study detected the target parasite.

### 2.8. Risk of Bias Assessment

The methodological quality of included studies was assessed using the Quality Assessment of Diagnostic Accuracy Studies-2 (QUADAS-2) tool and the Joanna Briggs Institute (JBI) critical appraisal checklist for diagnostic accuracy studies [13]. Four domains were evaluated: patient selection, index test, reference standard, and flow and timing. Each domain was assessed for risk of bias and categorized as low, high, or unclear risk according to the QUADAS-2 guidance document. Two authors (M.K., J.J.) independently performed the quality assessment, and disagreements were resolved through discussion.

### 2.9. Statistical Analysis

Meta-analyses were conducted to obtain pooled estimates of diagnostic sensitivity and corresponding 95% confidence intervals (95% CIs). Sensitivity was calculated as the proportion of true-positive cases identified by Mini Parasep^®^ among all positive cases detected by the composite reference standard. A random-effects model (DerSimonian–Laird) [14] was applied to account for expected methodological and clinical heterogeneity among studies. Statistical heterogeneity was assessed using Cochran’s Q test and quantified using the *I*^2^ statistic. Values of approximately 25%, 50%, and 75% were interpreted as indicating low, moderate, and high heterogeneity [15], respectively.

Subgroup analyses were performed according to study design (cross-sectional, diagnostic accuracy, and prospective studies), geographic region (Asia and Africa), country, and participant characteristics. Participant subgroups included community-based populations, schoolchildren, migrant workers, antenatal clinic patients, hospital samples, and specimens obtained from parasitology laboratories. Separate pooled sensitivity estimates were generated for each subgroup where sufficient data were available.

Results are presented as pooled sensitivity estimates with 95% confidence intervals (CIs). Between-study heterogeneity was evaluated and reported alongside pooled effect estimates. Publication bias was assessed using funnel plots when at least 10 studies were available for a given meta-analysis [16]. Statistical analyses were conducted using RStudio (Version 2024.04.2+764) [17], and statistical significance was defined as a two-sided *p*-value < 0.05.

Use of generative AI. During the preparation of this manuscript, ChatGPT (OpenAI, San Francisco, CA, USA) was used solely for language editing to improve the grammar, readability, and clarity of the manuscript. The tool was not used for study design, data collection, data analysis, interpretation of the results, or the generation of scientific conclusions. All AI-generated suggestions were critically reviewed, verified, and edited by the authors.

## 3. Results

### 3.1. Search Results

A total of 552 records were identified through main database searches, and 200 records through Google Scholar. After removing 67 duplicate records, 485 records from the main databases were screened based on titles and abstracts, of which 41 reports were assessed for eligibility. Of these, 34 reports were excluded for specific reasons, including incomplete data for analysis, conference abstracts without full-text availability, and the absence of a composite reference standard. For the Google Scholar search, 200 records were screened, and 12 reports were assessed for eligibility; of these, four met the inclusion criteria. Finally, 11 studies [8,10,11,12,18,19,20,21,22,23,24] met the eligibility criteria and were included in the systematic review and meta-analysis (Figure 1).

### 3.2. Characteristics of Included Studies

A total of 11 studies were included in the systematic review and meta-analysis; most studies were conducted in Asia (8/11, 72.7%), while the remaining studies were conducted in Africa (3/11, 27.3%). Thailand contributed the largest number of studies (7/11, 63.6%), followed by Ethiopia (1/11, 9.1%), Nigeria (1/11, 9.1%), Kenya (1/11, 9.1%), and India (1/11, 9.1%). Regarding study design, cross-sectional studies were the most frequent (6/11, 54.5%), followed by diagnostic accuracy studies (4/11, 36.4%) and one prospective study (1/11, 9.1%). Participants included among 11 studies were community participants (4/11, 36.4%), schoolchildren (2/11, 18.2%), migrant workers (1/11, 9.1%), antenatal clinic patients (1/11, 9.1%), hospital samples (1/11, 9.1%), and samples from the university (1/11, 9.1%). One study evaluated both community participants and schoolchildren (1/11, 9.1%). Details of included studies are demonstrated in Table S2.

### 3.3. Risk of Bias Assessment Results

Quality assessment using QUADAS-2 demonstrated that the Flow and Timing domain had the lowest risk of bias (10/11, 90.9%, Table 1). The Reference Standard domain was judged to be at high risk of bias (7/11, 63.6%) due to the use of composite reference standards that included the index test results. The Index Test domain was predominantly rated as unclear (81.8%, 9/11) because information regarding blinding of test interpretation was insufficiently reported. The Patient Selection domain was frequently assessed as unclear (7/11, 63.6%) due to inadequate descriptions of sampling procedures. Only one study (1/11, 9.1%) was judged to have a high risk of bias in patient selection because of its case-enriched design involving known positive specimens.

### 3.4. Detection of Any Intestinal Parasite

Overall, Mini Parasep^®^ demonstrated good diagnostic performance for the detection of any intestinal parasite, with a pooled sensitivity of 82.4% (95% CI: 61.0–93.4%, *I*^2^ = 95.3%, 6 studies, Table 2). Subgroup analysis showed that the highest sensitivity was observed in the single prospective study (99.4%, 95% CI: 90.9–100.0%). Diagnostic accuracy studies reported a pooled sensitivity of 78.8% (95% CI: 31.2–96.8%, *I*^2^ = 76.0%, 2 studies), while cross-sectional studies reported a sensitivity of 76.2% (95% CI: 50.8–90.9%, *I*^2^ = 96.1%, 3 studies).

Mini Parasep^®^ had a higher pooled sensitivity in African studies than in Asian studies. The pooled sensitivity in Africa was 90.4% (95% CI: 86.8–93.1%, *I*^2^ = 0%, 2 studies). In contrast, studies conducted in Asia reported a lower pooled sensitivity of 77.8% (95% CI: 42.0–94.4%, *I*^2^ = 80.9%, 4 studies).

Subgroup analysis by country revealed that the highest sensitivity was reported in India (99.4%, 95% CI: 90.9–100.0%), followed by Nigeria (93.3%, 95% CI: 64.8–99.1%, 1 study) and Ethiopia (90.2%, 95% CI: 86.5–93.0%, 1 study). In contrast, studies from Thailand reported a substantially lower pooled sensitivity of 63.5% (95% CI: 53.1–72.7%, *I*^2^ = 63.4%, 3 studies).

Diagnostic sensitivity also showed that the highest sensitivity was observed in samples obtained from a Department of Parasitology (99.4%, 95% CI: 90.9–100.0%, 1 study). Among schoolchildren, the pooled sensitivity was 77.6% (95% CI: 33.5–96.0%, *I*^2^ = 98.0%, 2 studies). Community-based studies showed the lowest pooled sensitivity (67.2%, 95% CI: 54.2–77.9%, *I*^2^ = 63.5%, 2 studies).

### 3.5. Pathogenic Protozoa

For detecting *E. histolytica*, Mini Parasep^®^ demonstrated a pooled sensitivity of 78.9% (95% CI: 39.3–95.6%, *I*^2^ = 43.7%, 4 studies). Subgroup analysis suggested that the sensitivity was higher in diagnostic accuracy studies (88.0%, *I*^2^ = 0%, 2 studies) than in cross-sectional studies (73.4%, *I*^2^ = 71.9%, 2 studies). Sensitivity was higher in African studies (94.1%, *I*^2^ = 0.0%, 2 studies) than in Asian studies (48.8%, *I*^2^ = 0.0%, 2 studies). Subgroup analysis by participant group showed that the highest sensitivity was observed among antenatal clinic patients (93.8%, 1 study), followed by hospital samples (75.0%, 1 study) and schoolchildren (73.4%, 2 studies).

Mini Parasep^®^ showed a pooled sensitivity of 76.3% (95% CI: 34.8–95.1%, *I*^2^ = 71.1%, 4 studies) for detecting *G. duodenalis*. Subgroup analysis suggested that the sensitivity appeared substantially higher in African studies (87.1%, 2 studies) than in Asian studies (69.4%, 2 studies). Diagnostic accuracy studies showed that the sensitivity appeared substantially higher in diagnostic accuracy studies (90.6%, *I*^2^ = 0.0%, 2 studies) than in cross-sectional studies (60.3%, *I*^2^ = 69.8%, 2 studies). Subgroup analysis by participants suggested that Mini Parasep^®^ demonstrated a highest sensitivity for samples from a hospital (92.9%, 1 study), followed by antenatal clinic patients (83.3%, 1 study), and schoolchildren (60.3%, 2 studies). For *Blastocystis* spp., Mini Parasep^®^ achieved a pooled sensitivity of 74.4% (95% CI: 60.4–84.7%, *I*^2^ = 0%, 2 studies).

### 3.6. Soil-Transmitted Helminths

Mini Parasep^®^ showed relatively poor sensitivity for hookworm detection, with a pooled estimate of 53.6% (95% CI: 33.3–72.8%, *I*^2^ = 23.1%, 5 studies). Subgroup analysis suggested that the sensitivity appeared substantially higher in diagnostic accuracy studies (72.7%, 2 studies) than cross-sectional studies (43.3%, 3 studies). Subgroup analysis by continent showed higher sensitivity in Asian studies (63.7%, 3 studies) than African studies (48.8%, 2 studies). Subgroup analysis by participants suggested that Mini Parasep^®^ demonstrated the highest sensitivity among migrant workers (83.3%, 1 study), followed by antenatal clinic patients (75.0%, 1 study), samples from a hospital (71.4%, 1 study), and lowest sensitivity in schoolchildren (41.9%, 2 studies).

For *A. lumbricoides*, Mini Parasep^®^ demonstrated a pooled sensitivity of 92.8% (95% CI: 88.4–95.7%, *I*^2^ = 0%, 2 studies). The pooled sensitivity of Mini Parasep^®^ for *T. trichiura* was 39.4% (95% CI: 8.6–81.8%; *I*^2^ = 74.8%; 3 studies). Mini Parasep^®^ achieved a pooled sensitivity of 73.7% (95% CI: 50.2–88.6%; *I*^2^ = 0%; 3 studies) for detecting *S. stercoralis*.

### 3.7. Tapeworms

For *Taenia* spp., Mini Parasep^®^ demonstrated a high pooled sensitivity of 89.0% (95% CI: 74.0–95.8%; *I*^2^ = 0%; 3 studies).

### 3.8. Flukes

For *O. viverrini*, Mini Parasep^®^ demonstrated a pooled sensitivity of 56.8% (95% CI: 35.1–76.1%, *I*^2^ = 88.8%, 7 studies). Subgroup analysis of study design suggested that cross-sectional studies reported a pooled sensitivity of 57.3% (95% CI: 29.2–81.3%, *I*^2^ = 91.8%, 4 studies), while diagnostic accuracy studies reported a slightly higher sensitivity of 60.1% (95% CI: 18.8–90.8%, *I*^2^ = 85.4%, 3 studies). All included studies were conducted in Thailand; therefore, the country-specific estimate is identical to the overall pooled estimate (56.8%, 95% CI: 35.1–76.1%; *I*^2^ = 88.8%). Subgroup analysis of participant characteristics showed that the lowest sensitivity was observed among schoolchildren (16.7%, 95% CI: 0.9–80.6%, 1 study). Studies conducted in community participants reported a pooled sensitivity of 44.7% (95% CI: 29.9–60.4%, *I*^2^ = 87.1%, 4 studies). In contrast, sensitivity was substantially higher among migrant workers (82.5%, 95% CI: 67.6–91.4%, 1 study) and was highest in hospital-based samples (94.1%, 95% CI: 68.0–99.2%, 1 study). For *S. mansoni*, Mini Parasep^®^ demonstrated a pooled sensitivity of 92.8% (95% CI: 86.9–96.1%, *I*^2^ = 0%, 2 studies).

## 4. Discussion

This systematic review and meta-analysis provides the first quantitative synthesis of the diagnostic sensitivity of Mini Parasep^®^ for the detection of intestinal parasites across multiple parasite groups. Based on 11 studies, Mini Parasep^®^ demonstrated good overall sensitivity (>80%) for detecting intestinal parasitic infections, although diagnostic performance varied according to parasite species, study design, study setting, and participant characteristics.

The pooled sensitivity of 82.4% for detecting any intestinal parasite indicated that Mini Parasep^®^ can detect the majority of infections when compared with composite reference standards. This level of sensitivity is encouraging because Mini Parasep^®^ offers practical advantages over conventional concentration techniques, including a closed processing system, reduced exposure of laboratory personnel to hazardous reagents, standardized specimen handling, and simplified workflow [10,11,18]. These characteristics may improve laboratory safety and operational efficiency, particularly in routine diagnostic laboratories and field-based epidemiological surveys.

Subgroup analyses suggested some differences according to study setting and participant characteristics. Studies conducted in Africa (90.4%) generally reported higher sensitivities than studies conducted in Asia (77.8%), particularly for overall parasite detection and pathogenic protozoa. These geographical differences should be interpreted cautiously because they are likely influenced by differences in parasite epidemiology, infection intensity, specimen preservation procedures, laboratory expertise, and reference standards rather than inherent differences in the diagnostic performance of Mini Parasep^®^. This finding highlights the importance of validating diagnostic methods under local epidemiological and laboratory conditions before widespread implementation.

The diagnostic sensitivity of Mini Parasep^®^ varied among protozoa. Relatively high pooled sensitivities were observed for *E. histolytica* (78.9%), *G. duodenalis* (76.3%), and *Blastocystis* spp. (74.4%). These results indicated that the concentration process effectively recovers protozoan cysts and trophozoites from fecal specimens. Higher sensitivities observed in diagnostic accuracy studies may reflect improved specimen handling, standardized microscopy procedures, or higher parasite burdens compared with community-based surveys. Interestingly, studies conducted in Africa generally reported higher sensitivities than those conducted in Asia. This difference may be attributable to variations in parasite burden, specimen preservation procedures, or differences in study populations. However, because the number of studies was limited, these regional differences should be interpreted cautiously.

Among STHs, Mini Parasep^®^ showed excellent sensitivity for *A. lumbricoides* (92.8%), but considerably lower sensitivity for hookworm (53.6%) and *T. trichiura* (39.4%). The high sensitivity for *A. lumbricoides* is likely attributable to the large size and robust morphology of its eggs, which facilitate recovery and microscopic identification. In contrast, hookworm eggs are relatively fragile and may degenerate during specimen storage or processing, potentially reducing detection rates [25,26]. The poor sensitivity observed for *T. trichiura* may be related to lower egg-recovery efficiency and variation in infection intensity across study populations. Previous evidence suggests substantial geographical variation in the intensity of *T. trichiura* infection, with a meta-analysis reporting lower prevalences of moderate-(0.62%) and high-intensity (0.01%) infections in sub-Saharan Africa compared with South-Eastern Asia, where corresponding prevalences were 4.43% and 0.73%, respectively [27]. Given that two of the studies included in the present analysis were conducted in Africa [10,20], the lower infection intensity in these settings may have contributed to the reduced sensitivity observed for *T. trichiura*. However, this interpretation should be approached with caution, as infection intensity data were not consistently reported across all included studies.

For *S. stercoralis* larvae, Mini Parasep^®^ demonstrated moderate sensitivity (73.7%). Although this performance is higher than that observed for other helminths, such as hookworm (53.6%) and *T. trichiura* (39.4%), its sensitivity remains moderate because the detection of *S. stercoralis* relies on identifying larvae rather than eggs, and larvae are often shed intermittently and in low numbers [28,29], making them difficult to detect by conventional stool microscopy. Specialized methods, such as agar plate culture [30] or molecular techniques [31,32], may provide superior sensitivity for detecting *S. stercoralis* infections.

The performance of Mini Parasep^®^ for flukes varied by parasite species. Sensitivity was very high for *S. mansoni* (92.8%) but only moderate for *O. viverrini* (56.8%). The high sensitivity observed for *S. mansoni* may be explained by the relatively large size and distinctive morphology of schistosome eggs, which facilitate their recovery and identification following concentration procedures. In contrast, the moderate sensitivity observed for *O. viverrini* is likely attributable to the small size of the eggs and the generally low egg output in infected individuals [12], both of which may reduce the probability of detection during microscopic examination.

Mini Parasep^®^ demonstrated high sensitivity for *Taenia* spp. (89.0%), likely because the relatively large and morphologically distinctive eggs are readily recovered and identified during microscopic examination. However, this estimate was derived from a limited number of studies and positive cases and should therefore be interpreted with caution. Additional studies with larger sample sizes are needed to further evaluate and confirm the diagnostic performance of Mini Parasep^®^ for tapeworm infections.

Several limitations should be considered when interpreting findings from this study. First, most included studies used a composite reference standard based on positive findings from multiple microscopic techniques. Because most included studies used a composite reference standard incorporating the index test, false-positive results were absent in nearly all studies, resulting in uniformly high specificity. Therefore, the summary receiver operating characteristic (SROC) curve should be interpreted cautiously, as it primarily reflects variation in sensitivity rather than the full trade-off between sensitivity and specificity. Future studies using independent molecular or antigen-based reference methods would provide more robust estimates of diagnostic accuracy. Second, substantial statistical heterogeneity was observed in several pooled analyses, particularly for overall parasite detection, *T. trichiura*, and *O. viverrini* infections. Substantial heterogeneity was observed across several pooled analyses because the included studies differed in geographical settings, study populations, infection intensities, stool preservation methods, reference standards, and laboratory procedures. In addition, the sensitivity of microscopic techniques is strongly influenced by infection intensity, which may contribute to variability in findings across studies. Consequently, pooled estimates should be interpreted as average performance across diverse settings rather than fixed measures of diagnostic accuracy. Third, the number of studies available for several parasite species was limited, reducing the precision of subgroup estimates. Finally, most studies included in the meta-analysis originated from Thailand and other Asian countries, potentially limiting the generalizability of the findings to other endemic regions.

The findings of this study have important implications for both clinical practice and public health. The variation in diagnostic sensitivity across parasite species highlights the importance of selecting diagnostic methods that are appropriate for the biological characteristics of the target organism. Mini Parasep^®^ performed particularly well for parasites producing large, morphologically distinctive eggs, whereas its sensitivity was lower for organisms characterized by low egg output, intermittent larval shedding, or fragile developmental stages. These differences are likely influenced not only by parasite biology but also by host-related factors, such as infection intensity and immune status, as well as laboratory factors including specimen preservation, microscopy quality, and technician expertise. Consequently, Mini Parasep^®^ appears to be well suited for routine diagnostic laboratories, epidemiological surveys, and surveillance programmes where standardized specimen processing, laboratory safety, and operational efficiency are priorities. However, complementary diagnostic methods may be required when highly sensitive detection of low-intensity infections is essential.

Beyond its diagnostic performance, Mini Parasep^®^ offers broader advantages through its standardized closed-system workflow, which reduces exposure to hazardous reagents while improving consistency in stool processing. These characteristics make it an attractive option for laboratories with varying levels of infrastructure and may facilitate harmonization of diagnostic procedures across surveillance networks. Such standardization may improve the comparability of prevalence estimates across surveillance programs and strengthen evidence used to guide parasite control strategies. In resource-limited settings, Mini Parasep^®^ may represent a practical alternative to conventional concentration techniques because of its simplified workflow and reduced biosafety concerns, although future studies evaluating cost-effectiveness and implementation feasibility in different healthcare settings are warranted.

Future research should focus on validating Mini Parasep^®^ using independent reference standards, including molecular assays such as polymerase chain reaction (PCR), quantitative PCR (qPCR), or loop-mediated isothermal amplification (LAMP), to better define its diagnostic accuracy, particularly for low-intensity infections. Hybrid diagnostic workflows that combine Mini Parasep^®^ as a standardized concentration method with molecular detection may improve sensitivity while maintaining the practical advantages of specimen concentration. In addition, future multicenter studies conducted across different endemic regions should evaluate diagnostic performance under diverse epidemiological conditions and laboratory capacities to establish context-specific recommendations for implementation. Emerging technologies, including artificial intelligence-assisted image analysis, may further improve the reproducibility and efficiency of parasite identification when integrated with standardized stool concentration methods such as Mini Parasep^®^. Furthermore, because the device provides a standardized method for concentrating fecal specimens, its potential application within a One Health framework—including surveillance of zoonotic parasites in animal and environmental samples—merits further investigation.

## 5. Conclusions

Mini Parasep^®^ demonstrated good overall sensitivity for detecting intestinal parasites and performed particularly well for *A. lumbricoides*, *S. mansoni*, and *Taenia* spp. However, reduced sensitivity for *T. trichiura*, hookworm, and *O. viverrini* indicates that diagnostic performance remains species-dependent. Although Mini Parasep^®^ offers operational advantages and may be suitable for routine screening and epidemiological surveys, additional high-quality studies using independent reference standards are required to further define its diagnostic accuracy.

## Figures and Tables

**Table 1:** QUADAS-2 assessment.

Study	Patient Selection	Index Test	Reference Standard	Flow & Timing
Abdulkanan et al., 2022 [18]	Low	Unclear	Unclear	Low
Adugna et al., 2017 [10]	Low	Unclear	Unclear	Low
Charoensuk et al., 2019 [19]	Unclear	Low	Low	Low
Ikeh et al., 2021 [20]	High	Unclear	High	Low
Kaewpitoon et al., 2017 [21]	Unclear	Unclear	Unclear	Low
Kopolrat et al., 2022 [22]	Low	Unclear	High	Low
Laoprom et al., 2016 [23]	Unclear	Unclear	High	Low
Mewara et al., 2019 [11]	Unclear	Low	High	Low
Ng’etich et al., 2016 [24]	Unclear	Unclear	High	High
Prakobwong et al., 2024 [12]	Unclear	Unclear	High	Low
Sanprasert et al., 2016 [8]	Unclear	Unclear	High	Low

**Table 2:** Sensitivity of Mini Parasep^®^ for detecting intestinal parasites compared to the composite reference standard.

Parasite Species	Subgroup Analysis	Pooled Sensitivity (95% CI) (%)	*I*^2^ (%)	N. of Events	N. of Observation	N. Studies (Subsets)	References
**Any intestinal parasitic infections**	**Overall**	82.42 (61.04–93.35)	95.3	553	681	6	[8,10,11,20,22,23]
	**Study design**						
	Cross-sectional study	76.24 (50.81–90.88)	96.1			3	
	Diagnostic accuracy	78.75 (31.22–96.80)	76.0			2	
	Prospective study	99.38 (90.90–99.96)	N/A			1	
	**Continent**						
	Africa	90.36 (86.75–93.06)	0.0			2	
	Asia	77.81 (42.04; 94.43)	80.9			4	
	Mixed (multi-continent)						
	**Country**						
	Thailand	63.47 (53.08–72.74)	63.4			3	
	Ethiopia	90.24 (86.53–93.02)	N/A			1	
	Nigeria	93.33 (64.80–99.07)	N/A			1	
	India	99.38 (90.90–99.96)	N/A			1	
	**Participants**						
	Schoolchildren	77.61 (33.50–95.97)	98.0			2	
	Participants in community	67.15 (54.23–77.91)	63.5			2	
	Samples from the Department of Parasitology	99.38 (90.90–99.96)	N/A			1	
	Antenatal clinic patients	93.33 (64.80–99.07)	N/A			1	
**Pathogenic protozoa**							
***E. histolytica***	**Overall**	78.91 (39.31–95.58)	43.7	18	21	4	[8,10,18,20]
	**Study design**						
	Cross-sectional study	73.41 (10.58–98.47)	71.9			2	
	Diagnostic accuracy	88.00 (46.48–98.41)	0.0			2	
	**Continent**						
	Africa	94.11 (67.93–99.18)	0.0			2	
	Asia	48.82 (16.67–81.97)	0.0			2	
	**Country**						
	Thailand	48.82 (16.67–81.97)	0.0			2	
	Ethiopia	94.44 (49.53–99.66)	N/A			1	
	Nigeria	93.75 (46.14–99.62)	N/A			1	
	**Participants**						
	Schoolchildren	73.41 (10.58–98.47)	71.9			2	
	Antenatal clinic patients	93.75 (46.14–99.62)	N/A			1	
	Samples from a hospital	75.00 (10.89–98.66)	N/A			1	
***Blastocystis*** **spp.**	**Overall**	74.40 (60.44–84.69)	0.0	36	48	2	[8,18]
***G. duodenalis***	**Overall**	76.25 (34.78–95.08)	71.1	24	35	4	[8,10,18,20]
	**Study design**						
	Cross-sectional study	60.34 (8.81–95.99)	69.8			2	
	Diagnostic accuracy	90.63 (64.09–98.13)	0.0			2	
	**Continent**						
	Africa	87.15 (45.25–98.24)	0.0			2	
	Asia	69.35 (8.57–98.20)	87.0			2	
	**Country**						
	Thailand	69.35 (8.57–98.20)	87.0			2	
	Ethiopia	90.00 (32.64–99.41)	N/A			1	
	Nigeria	83.33 (19.36–99.05)	N/A			1	
	**Participants**						
	Schoolchildren	60.34 (8.81–95.99)	69.8			2	
	Samples from a hospital	92.86 (62.97–99.00)	N/A			1	
	Antenatal clinic patients	83.33 (19.36–99.05)	N/A			1	
**Soil-transmitted helminths**							
***A. lumbricoides***	**Overall**	92.83 (88.35–95.67)	0.0	188	202	2	[10,18]
**Hookworm**	**Overall**	53.56 (33.25–72.75)	23.1	34	71	5	[8,10,18,20,21]
	**Study design**						
	Cross-sectional study	43.26 (31.32–56.04)	10.1			3	
	Diagnostic accuracy	72.69 (41.37–90.95)	0.0			2	
	**Continent**						
	Africa	48.77 (25.45–72.64)	26.8			2	
	Asia	63.65 (30.92–87.26)	23.1			3	
	**Country**						
	Thailand	63.65 (30.92–87.26)	23.1			3	
	Ethiopia	42.86 (30.64–56.01)	N/A			1	
	Nigeria	75.00 (23.78–96.65)	N/A			1	
	**Participants**						
	Schoolchildren	41.91 (29.98–54.85)	0.0			2	
	Samples from a hospital	71.43 (32.66–92.80)	N/A			1	
	Antenatal clinic patients	75.00 (23.78–96.65)	N/A			1	
	Migrant workers	83.33 (19.36–99.05)	N/A			1	
***T. trichiura***	**Overall**	39.42 (8.61–81.81)	74.8	144	214	3	[10,18,20]
***S. stercoralis***	**Overall**	73.68 (50.20–88.60)	0.0	13	17	3	[10,18,21]
**Tapeworms**							
***Taenia*** **spp.**	**Overall**	89.00 (74.01–95.83)	0.0	36	39	3	[10,18,21]
**Flukes**							
***O. viverrini***	**Overall**	56.78 (35.13–76.11)	88.8	225	498	7	[8,12,18,19,21,22,23]
	**Study design**						
	Cross-sectional study	57.25 (29.23–81.29)	91.8			4	
	Diagnostic accuracy	60.12 (18.78–90.77)	85.4			3	
	**Continent/Country**						
	Thailand	56.78 (35.13–76.11)	88.8			7	
	**Participants**						
	Participants in communities	44.68 (29.94–60.43)	87.1			4	
	Schoolchildren	16.67 (0.95–80.64)	N/A			1	
	Migrant workers	82.50 (67.59–91.42)	N/A			1	
	Samples from a hospital	94.12 (67.97–99.18)	N/A			1	
***S. mansoni***	**Overall**	92.77 (86.88–96.13)	0.0	123	132	2	[10,20]

Abbreviations: N/A: Not assessed.

## Data Availability

All data related to the present study are available in this manuscript, as well as in the Tables S1 and S2 files.

## Supplementary Materials

The following supporting information can be downloaded at: https://www.mdpi.com/article/10.3390/medicina62081464/s1, Table S1: Search strategy; Table S2: Details of included studies.
