# Supplementary material for: Diagnostic Performance of Mini Parasep® for the Detection of Intestinal Parasitic Infections: A Systematic Review and Meta-Analysis
Source: Medicina (Kaunas). 2026 Jul 28;62(8):1464. doi: 10.3390/medicina62081464 (PMC13514642; doi:10.3390/medicina62081464)
Supplement: Supplementary file 1 [file medicina-62-01464-s001.zip › Table S1. Search terms.pdf]

Table S1. Search terms

General keywords

"Mini Parasep®" OR "Mini Parasep® solvent free" OR "Mini Parasep" OR “Mini Parasep SF” OR “Mini Parasep® SF Faecal Parasite Concentrator” OR “Mini Parasep® SF stool concentrator kit”

PubMed 20 April 2026

No.	Key concept	Search terms	Results
1.	Mini Parasep® SF stool concentrator kit	"Mini Parasep®"[MeSH Terms] OR "Mini Parasep® solvent free"[MeSH Terms] OR "Mini Parasep"[MeSH Terms] OR “Mini Parasep SF” [MeSH Terms] OR “Mini Parasep® SF Faecal Parasite Concentrator”[MeSH Terms] OR “Mini Parasep® SF stool concentrator kit”[MeSH Terms] OR "Mini Parasep®"[Text Word] OR "Mini Parasep® solvent free"[Text Word] OR "Mini Parasep"[Text Word] OR “Mini Parasep SF”[Text Word] OR “Mini Parasep® SF Faecal Parasite Concentrator”[Text Word] OR “Mini Parasep® SF stool concentrator kit”[Text Word]	25

Scopus 20 April 2026

No.	Key concept	Search terms	Results
1.	Mini Parasep® SF stool concentrator kit	TITLE-ABS-KEY ("Mini Parasep®" OR "Mini Parasep® solvent free" OR "Mini Parasep" OR "Mini Parasep SF" OR "Mini Parasep® SF Faecal Parasite Concentrator" OR "Mini Parasep® SF stool concentrator kit")	36

Ovid (Journal) 20 April 2026

No.	Key concept	Search terms	Results
1.	Mini Parasep® SF stool concentrator kit	"Mini Parasep®" OR "Mini Parasep® solvent free" OR "Mini Parasep" OR "Mini Parasep SF" OR "Mini Parasep® SF Faecal Parasite Concentrator" OR "Mini Parasep® SF stool concentrator kit" {Including Limited Related Terms}	431#

Nursing & Allied Health Premium 20 April 2026

No.	Key concept	Search terms	Results

1.	Mini Parasep® SF stool concentrator kit	"Mini Parasep®" OR "Mini Parasep® solvent free" OR "Mini Parasep" OR “Mini Parasep SF” OR “Mini Parasep® SF Faecal Parasite Concentrator” OR “Mini Parasep® SF stool concentrator kit”	14 #

Embase 20 April 2026

No.	Key concept	Search terms	Results
1.	Mini Parasep® SF stool concentrator kit	"Mini Parasep®"/exp OR "Mini Parasep® solvent free"/exp OR "Mini Parasep"/exp OR “Mini Parasep SF”/exp OR “Mini Parasep® SF Faecal Parasite Concentrator”/exp OR “Mini Parasep® SF stool concentrator kit”/exp OR "Mini Parasep®":ti,ab,kw OR "Mini Parasep® solvent free":ti,ab,kw OR "Mini Parasep":ti,ab,kw OR “Mini Parasep SF”:ti,ab,kw OR “Mini Parasep® SF Faecal Parasite Concentrator”:ti,ab,kw OR “Mini Parasep® SF stool concentrator kit”:ti,ab,kw	46

Google Scholar 20 April 2026

No.	Key concept	Search terms	Results
1.	Mini Parasep® SF stool concentrator kit	"Mini Parasep®" OR "Mini Parasep® solvent free" OR "Mini Parasep" OR “Mini Parasep SF” OR “Mini Parasep® SF Faecal Parasite Concentrator” OR “Mini Parasep® SF stool concentrator kit”	356 (Screening for the first 200 results)

Google Scholar (n = 200);

154 Un-related articles

46 related articles were screened and 34 were excluded with reasons;

- Duplicated with articles retrieved from main databases (n = 29)
- Thesis without full data (n = 2)
- Reviews (n = 2)
- Conference abstract (n = 1)

Potentially included (n = 12)

- Incomplete data for calculating the pooled sensitivity (n = 5)
- Animal samples (n = 1)
- Not Mini Parasep® kit (n = 1)
- Not composite reference standard (n = 1)

- Final included (n = 4)

Thai-Journal Citation Index Centre (TCI) 19 May 2026

No.	Key concept	Search terms	Results
1.	Mini Parasep® SF stool concentrator kit	Mini Parasep	5

- Incomplete data (n = 2)
- Duplicated with articles selected in Google Scholar (n = 2)
- Duplicated participant groups with the article selected from the main databases (n = 1)#
